# Post-Anaphylaxis Adrenaline-Induced Takotsubo Cardiomyopathy: A Case Report

**DOI:** 10.7759/cureus.84593

**Published:** 2025-05-22

**Authors:** Zahid Khan, Fady Eldabe, Konstantinos Tyrovolas, Krishna Rathod, Paul Rees

**Affiliations:** 1 Acute Medicine, Mid and South Essex NHS Foundation Trust, Southend-on-Sea, GBR; 2 Cardiology, Barts Heart Centre, London, GBR; 3 Cardiology and General Medicine, Barking, Havering and Redbridge University Hospitals NHS Trust, London, GBR; 4 Cardiology, Royal Free Hospital, London, GBR; 5 Cardiology, Queen Mary University of London, London, GBR

**Keywords:** adrenaline autoinjector, adrenaline induced takosubo cardiomyopathy, allergy to food, drug induced anaphylaxis, lv gram, normal coronary angiogram, pistachio induced anaphylaxis, st-elevation myocardial infarction (stemi), takotsubo cardioyopathy, trans thoracic echocardiography

## Abstract

Takotsubo cardiomyopathy (TCM) is an acute and reversible cardiac condition triggered by an adrenaline rush in response to stress that is characterised by apical ballooning of the left ventricle in the absence of coronary artery obstruction. Although the exact pathophysiology remains unclear, it is believed to be secondary to the release of adrenaline or catecholamine in response to stress. We present the case of a 71-year-old female who presented to a district general hospital (DGH) at approximately 1700 pm in the evening with signs and symptoms of anaphylaxis following the consumption of pistachios the night before. She woke up with mild shortness of breath (sob) in the morning at 0900 am. The symptoms progressively got worse after midday, and she developed throat tightness and mild tongue swelling at 1600, prompting her to attend the local hospital’s accident and emergency department. Following evaluation in the accident and emergency department, she was given 0.5 mg of adrenaline one in 1000 (1 mg/mL) solution intramuscularly for a delayed allergic reaction to pistachios. She developed central chest pain and tightness within minutes of receiving the intramuscular adrenaline, and an electrocardiogram showed ST elevation in the lateral leads and ST depression inferiorly. She was transferred to our cardiac centre for emergency coronary angiography, and bedside echocardiography revealed mild to moderate left ventricular systolic dysfunction (LVSD). Coronary angiography via the right radial access showed unobstructed coronaries. Left ventriculogram (LVG) post-angiogram demonstrated apical ballooning suggestive of TCM secondary to adrenaline administration for anaphylaxis to pistachio. Departmental echocardiography revealed a mild LVSD with an ejection fraction of 45%. She was started on bisoprolol in addition to her regular medications. Echocardiography demonstrated normal left ventricular function three months later, and the patient was discharged from the outpatient clinic.

## Introduction

Takotsubo cardiomyopathy (TCM), also known as broken heart syndrome or stress cardiomyopathy, is caused by intense emotional and physical stressors. The classical findings in patients with typical TCM include apical hypokinesia [1]. It typically mimics acute myocardial infarction (AMI) based on clinical presentation and electrocardiogram (ECG) findings in the absence of coronary artery disease (CAD) [2]. TCM is typically associated with emotional stress; however, it can also be caused by physical stress resulting from major illnesses, surgery, medical and obstetric, or psychiatric emergencies [3,4]. Patients with TCM can present with ECG changes suggestive of AMI and raised troponin T levels, and tend to have reversible transient LVSD [5]. The most common symptoms in patients with TCM include chest pain and dyspnea; Serious presentations such as ventricular fibrillation and cardiogenic shock are rare [5,6]. The ECG abnormalities, such as ST segment depression, elevation, T-wave inversion and left bundle branch block (LBBB), are mostly reversible and occur during the acute phase [4]. These patients usually have raised serum natriuretic peptide (brain natriuretic peptide (BNP) or N-terminal pro-B-type natriuretic peptide (NT-proBNP)) during the acute phase, and the left ventricular function mostly recovers within three to six months.

Case series from Asian and Western countries reported that approximately 1%-2.3% of those who presented with AMI-type presentation were diagnosed with TCM [5,6]. The two most significant Nationwide Inpatient Sample (NIS-USA) studies showed that TCM predominantly affected elderly post-menopausal women aged 66-80 years (90%), and the main risk factors were smoking, alcohol abuse, anxiety and hyperlipidaemia [7,8]. Other studies also showed that TCM was more common in postmenopausal women [9-11]. Three possible mechanisms may cause TCM: catecholamine-induced neurogenic cardiotoxicity, coronary microvascular impairment and multivessel epicardial coronary artery vasospasm [12]. The recurrence rate in patients with TCM is reported as 5-6% at five years in only a few patients, and there are only a few reported cases of recurrent TCM [13]. However, the Task Force on TCM 2015 reported that the prevalence varies from 5%-22% in hospital inpatients [9]. Only a few cases of adrenaline-induced TCM have been reported in the literature, and a systematic review reported 41 cases from 36 studies of patients receiving adrenaline administration resulting in TCM [14]. We present the case of a 71-year-old woman who presented with TCM secondary to adrenaline administration for anaphylaxis from the pistachio.

## Case presentation

A 71-year-old female presented to a district general hospital (DGH) at 1700 pm after waking up at 0900 am with shortness of breath (sob) initially. She had pistachios the night before at a restaurant. Her breathing progressively worsened from midday, and she noticed swelling in her eyes, lips and throat, and tightness and rash on her body at about 1600. At this point, she decided to attend the accident and emergency, and she was noted to have tongue swelling, swollen eyelids and wheeze on examination. She was tachycardic, tachypneic and hypotensive on clinical examination. She was given intravenous hydrocortisone 200 mg stat, intravenous 250 mL normal saline fluid bolus and 0.5 mg of one in 1000 adrenaline (1 mg/mL solution) intramuscularly for a delayed allergic reaction to pistachios. She developed central chest pain with diaphoresis within five minutes after adrenaline injection; ECG showed ST elevation in the lateral leads and ST depression in the inferior leads, and the patient was transferred to our cardiac centre for an emergency coronary angiogram. Emergency coronary angiography showed unobstructive coronary arteries, and the left ventriculogram (LVG) showed moderate LV dysfunction with apical ballooning and anterior wall hypokinesia (Videos 1-3). Echocardiography showed mild to moderate LV systolic dysfunction with a left ventricular ejection fraction of 45% and a hypokinetic apex, base/mid anterior wall, inferoseptal, and mid anteroseptal wall (Video 4). Past medical history (PMH) was significant for type 2 diabetes mellitus (T2DM), hypertension, depression, high cholesterol and diabetic retinopathy. Regular medications were amlodipine 10 mg once daily (OD), aspirin 75 mg OD, bisoprolol 2.5 mg OD, candesartan 32 mg OD, calcichew D3 twice daily (BD), metformin 1 g BD, pravastatin 40 mg OD, venlafaxine 150 mg modified release (MR) OD and dapagliflozin 10 mg OD. She was a lifelong non-smoker, non-drinker and self-caring.

**Video 1:** Coronary angiogram showing right coronary artery

**Video 2:** Coronary angiogram showing unobstructed left coronary system

**Video 3:** Left ventriculogram showing apical ballooning

**Video 4:** Echocardiogram with poor acoustic windows showing mildly impaired left ventricular function

**Figure 1:**
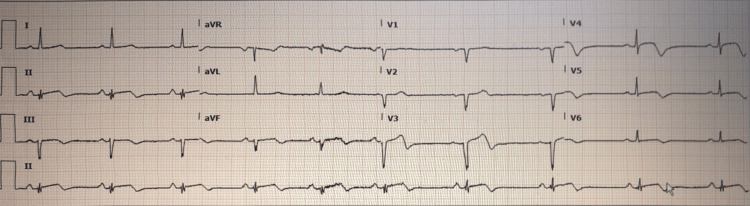
Electrocardiogram showing subtle ST elevation in leads II, III, and T wave inversion in V3-V6

Laboratory tests showed elevated troponin levels of 541 ng/L, peaking at 3529 (reference 0-14 ng/L). All other blood tests were normal except mildly raised bilirubin 32 (reference 0-21 μmol/L), alanine transaminase (ALT) level 38 (reference 0-32 unit/L), white cell count 10.3 (reference 4-10 × 109/L) and neutrophil count 7.9 (reference 2-7 × 109/L). Repeat ECG on the second day showed subtle ST elevation in the inferior leads and T-wave inversion in leads V3-V6 (Figure 1). She was initially treated with aspirin 300 mg stat and prasugrel 60 mg stat given suspected AMI, followed by aspirin 75 mg OD, prasugrel 10 mg OD and bisoprolol 2.5 mg OD. Both antiplatelet therapies were discontinued after a coronary angiogram revealed unobstructive coronaries, and the LVG demonstrated the likely cause to be TCM. She was discharged after two days of hospital stay and was booked for outpatient cardiac magnetic resonance imaging (CMR). CMR showed no evidence of myocardial infarction or myocarditis, confirming the diagnosis of TCM, and LV function improved to 69% and remained clinically stable (Video 5).

**Video 5:** Cardiac magnetic resonance imaging four-chamber view showing preserved left ventricular function with no regional wall motion abnormality or scarring

## Discussion

TCM, also known as stress cardiomyopathy, is caused by emotional and physical stress, as well as medications. Patients usually present with chest pain following an emotional or physical trigger, and an ECG may show ST-segment changes, including ST elevation and elevated troponin T or I. Echocardiography shows transient LVSD and apical ballooning; however, this may be difficult to elicit in some patients owing to poor acoustic windows. Left ventriculography and CMR can help confirm the diagnosis of TCM in these patients. It is more common in postmenopausal female patients, and most of these patients undergo coronary angiography, showing non-obstructive coronary arteries. TCM accounts for 0.7-2.5% of AMI cases, and although it is more common in post-menopausal women, it can also affect men and younger women to a lesser extent [3,4,9]. TCM tends to be more common in postmenopausal women aged > 50 years, whereas myocarditis tends to be common in younger patients and both sexes [4].

TCM generally has three distinct anatomical variants: apical with or without the mid-left ventricular (MLV) variant (typical), inverted or basal, and MLV, with a prevalence of 70-80%, 5%, and 10-15%, respectively [4]. Biventricular involvement is clinically less than 0.5 %; however, it is reported to be approximately 33% on CMR. The long-term prognosis is usually favourable, and most patients show complete recovery. Approximately 6-20% of patients may develop cardiogenic shock, whereas arrhythmia, including atrial fibrillation, is reported as 5-15%, and ventricular arrhythmias 4-9%, respectively [4]. A few patients may develop TCM during medical procedures or through medication administration [13,15]. In-hospital mortality is 1-4.5%, five-year mortality is 3-17%, and the recurrence mortality is 5-22% [4]. The European Heart Association Task Force advised categorising TCM into primary and secondary TCM based on clinical presentation. They further elaborated that patients with primary TCM present with cardiac symptoms. In contrast, patients with secondary TCM develop from receiving treatment for the primary condition, which could be surgical, medical or obstetric, resulting in catecholamine release leading to secondary TCM [4].

Ioannou published a case report of a patient who developed TCM secondary to erroneous administration of adrenaline in cardiac arrest, followed by return of spontaneous circulation (ROSC) after successful cardiopulmonary resuscitation (CPR) [1]. The patient had unobstructed coronary arteries on coronary angiography, and CMR demonstrated mild mid-anterior, inferior, lateral, and septal segment hypokinesia with mildly impaired left ventricular systolic function of 49%, which was resolved entirely on repeat CMR within three months. No regional wall motion abnormalities were identified on repeat CMR imaging. Spina et al. reported a case of a 68-year-old patient who developed secondary TCM due to subcutaneous administration of a higher dose of adrenaline by mistake in the cheek during induction of anaesthesia [3]. The patient developed ST-segment elevation, ventricular tachycardia, and elevated blood pressure that responded to medical therapy. Coronary angiography showed unobstructed coronary arteries, and the LVG showed apical ballooning and moderate systolic dysfunction, which recovered on repeat echocardiogram in one month. Bourenne et al. reported a case of a 28-year-old male patient who injected 5 mcg of intravenous adrenaline with suicidal intent and developed apical TC [16]. Belliveau et al. reported a case of TCM secondary to the administration of 1 mg adrenaline into the perineum with local anesthetic following vaginal delivery, resulting in TCM involving the mid-and basal segments [17].

Abraham et al. reported a case series of six patients developing TCM (three apical and three mid-ventricular/basal) due to the administration of adrenaline for surgical and non-surgical reasons. In one patient in this case series, self-injection of 40 mg of intravenous adrenaline resulted in apical TCM. All patients had moderate LVSD, which recovered after seven days at the follow-up visit [18]. Lagan et al. reported a case series of patients with typical and atypical TCM. They described typical TCM as involving apical ballooning and apical left ventricular region dyskinesis, whereas atypical TCM had features of mid-ventricular ballooning [5]. The LVG showed apical and mid-ventricular ballooning in these cases, and the patient had unobstructed coronary arteries. Our patient also had midventricular ballooning on an LVG, suggestive of atypical TCM with mildly impaired LV function, which recovered completely within three months.

## Conclusions

This case report highlights the importance of LVG in patients with TCM and raises awareness about the association between adrenaline administration and TCM, which is commonly used to treat anaphylaxis. Clinicians should be aware of this important but rarely observed clinical association. TCM has a good overall prognosis and most patients recover completely.
